# Enhancing semantic segmentation for autonomous vehicle scene understanding in indian context using modified CANet model

**DOI:** 10.1016/j.mex.2024.103131

**Published:** 2024-12-21

**Authors:** Smita Khairnar, Sudeep D. Thepade, Suresh Kolekar, Shilpa Gite, Biswajeet Pradhan, Abdullah Alamri, Bhagyesha Patil, Shrutee Dahake, Radhika Gaikwad, Atharva Chaudhari

**Affiliations:** a Department of Computer Engineering, Pimpri Chinchwad College of Engineering, Nigdi, Pune 411044, India; b Symbiosis Centre of Applied AI (SCAAI), Symbiosis International (Deemed) University, Pune 412115 India; c PCET's, Pimpri Chinchwad University, Pune, India; d Centre for Advanced Modelling and Geospatial Information Systems (CAMGIS), School of Civil and Environmental Engineering, University of Technology Sydney, NSW 2007, Australia; e Department of Geology and Geophysics, College of Science, King Saud University, Riyadh, Saudi Arabia; f Artificial Intelligence and Machine Learning Department, Symbiosis Institute of Technology, Symbiosis International (Deemed) University, Pune 412115, India

**Keywords:** CANet, Indian driving dataset lite, Link-net, Mean IoU, Scene understanding, U-Net, Autonomous Vehicle Scene Understanding in Indian Context Using Modified CANet Model

## Abstract

Recent advancements in artificial intelligence (AI) have increased interest in intelligent transportation systems, particularly autonomous vehicles. Safe navigation in traffic-heavy environments requires accurate road scene segmentation, yet traditional computer vision methods struggle with complex scenarios. This study emphasizes the role of deep learning in improving semantic segmentation using datasets like the Indian Driving Dataset (IDD), which presents unique challenges in chaotic road conditions. We propose a modified CANet that incorporates U-Net and LinkNet elements, focusing on accuracy, efficiency, and resilience. The CANet features an encoder-decoder architecture and a Multiscale Context Module (MCM) with three parallel branches to capture contextual information at multiple scales. Our experiments show that the proposed model achieves a mean Intersection over Union (mIoU) value of 0.7053, surpassing state-of-the-art models in efficiency and performance.

Here we demonstrate:

• Traditional computer vision methods struggle with complex driving scenarios, but deep learning based semantic segmentation methods show promising results.
• Modified CANet, incorporating U-Net and LinkNet elements is proposed for semantic segmentation of unstructured driving scenarios.
• The CANet structure consists of an encoder-decoder architecture and a Multiscale Context Module (MCM) with three parallel branches to capture contextual information at multiple scales.

## Graphical abstract

**Figure:**
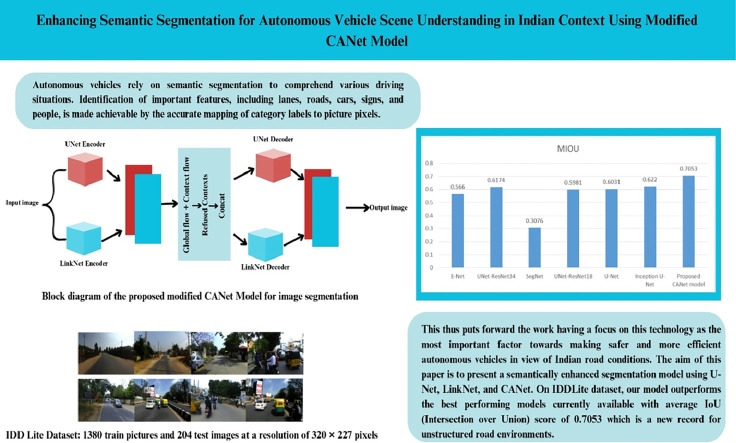

Specifications table

**Table:** 

Subject area:	Computer science
More specific subject area:	Image Processing.
Name of your method:	Autonomous Vehicle Scene Understanding in Indian Context Using Modified CANet Model
Name and reference of the original method:	N/A
Resource availability:	Indian Driving Dataset (IDD) https://idd.insaan.iiit.ac.in/dataset/details

## Introduction

With many vehicle kinds, a lack of lane discipline, and a disorganized pattern on key thoroughfares, India's highly congested and chaotic traffic conditions provide unique challenges for autonomous driving. The Indian Driving Dataset (IDD) Lite [1] aims to replicate the congested and unstructured urban road conditions typical of Indian streets. Accurate perception will be essential for exact scene parsing as autonomous vehicles start to navigate these busy roadways in the next few years. However, standard road segmentation algorithms must be revised since the expected structure has too many exceptions. Data-driven deep learning offers potential flexibility but requires large, diverse training datasets like those provided by IDD Lite.

Autonomous vehicles use semantic segmentation to comprehend various driving situations [2]. Essential characteristics, including roads, lanes, automobiles, signs, and people, maybe identified thanks to accurately mapping category names to image pixels. Making decisions and planning for navigation is aided by this pixel-level processing. Segmentation is challenging due to scene complexity, occlusion, size fluctuation, and other variables [1]. In India, conditions are further complicated by congested roads, erratic behaviour, diverse road users, varying lighting conditions, and weather. Customized approaches are necessary to address these specific challenges. In particular, densely populated locations and highways are covered by the Indian Driving Dataset (IDD) Lite benchmark, which includes a variety of situations. Before recently, segmentation algorithms relied on manually created features [1]; however, deep neural networks are now in charge when sufficient training data is available. Efficiency is achieved via progressive down sampling and up sampling balanced with lateral connections in UNet encoder-decoder systems [3], such as LinkNet, which provide a multiscale context.

Numerous methods, strategies, and frameworks could be used to address various deep learning problems. The CANet model is a proposed design that addresses the issue of picture segmentation. This method leverages gathering multiscale contexts with a U-Net-related fully convolutional network to obtain precise localization and image segmentation masks. The proposed model proposes a series-parallel composite approach using the CAM (Chained Context Aggregation Module) to diversify feature transmission [4].

One of CANet's primary contributions is the Chained Context Aggregation module [5], which combines parallel and multitudinous Hybrid Structures to collect diverse layers of information. While parallel flows encode distinct region-based contexts and significantly improve performance, serial flows gradually expand the receptive areas of output neurons. Flow guiding enables potent multiscale context aggregation [4]. The model delivers optimal results following comprehensive testing on numerous challenging datasets, positioning it as a state-of-the-art performance technique.

This study makes several significant contributions. Firstly, it proposes adopting the CANet architecture, which integrates U-Net and Link-Net models. This architecture offers an efficient solution for precise semantic segmentation, crucial for autonomous vehicle navigation, especially in diverse and unpredictable Indian road conditions. Secondly, the study evaluates the modified CANet architecture's effectiveness compared to existing semantic segmentation models. Finally, it validates the performance of the proposed CANet model and existing semantic segmentation models using the mIoU performance metric, leveraging the IDD-Lite dataset. Overall, these contributions highlight the potential of the CANet architecture in enhancing semantic segmentation for autonomous vehicle navigation in challenging real-world scenarios. Our new approach represents a modified version of CANet architecture. The convolutional layers of the basic CANet model are replaced by encoders and decoders of the LinkNet and Unet models, consisting of convolutional layers and blocks. This increased the value of the mean IoU performance metric, which validates the execution of models for semantic segmentation. To assess the performance of our suggested model, we compared it with existing models such as ResNet18, U-Net, E-Net, SegNet, and UNet-ResNet3. Our suggested model achieved a mean IoU value of 0.7053, which is higher than previous models of similar kinds.

## Background

An essential element in visual processing is semantic segmentation [2]. It involves grouping the image into parts which make sense semantically and labelling each pixel with the name of an object or class to which it belongs. Advances in deep learning techniques, particularly with models based on convolutional networks, have led to a significant evolution over the last ten years. This literature research review presents a comprehensive overview of current developments and methods for classification, such as techniques that differ from traditional approaches to the state of deep learning systems.

Tang et al. [4] tackled the issue of complicated scene semantic segmentation by proposing a novel method to leverage the attention-guided chained context. To identify the shortcomings of existing semantic segmentation techniques, especially when dealing with complex scenarios involving a variety of objects and backgrounds, an extensive literature review is carried out by the authors. They described the drawbacks of typical approaches for seamlessly capturing contextual data in various spatial resolutions. Drawing upon the recent development in deep learning technology and attention mechanisms, the work of Tang et al. adopts a focus-oriented model alternative to the previously mentioned known attention models, which have previously only been used in other kinds of tasks. Integrating focus-oriented mechanisms into a coherent, structured context aggregation structure significantly helps promote the model's capacity to obtain the long-range support and context hints essential to proper scene interpretation. Tang et al. motivated the proposed approach and provided a solid foundation for the model design to address the challenges of complex semantic segmentation tasks.

Siam et al. [6] evaluated the performance of the current offerings in semantic segmentation for autonomously operated scenarios. Reviewing traditional computer vision and deep learning techniques, the authors assessed the strengths and limitations in handling the complexities of driving-related situations and computational constraints. Their review paper provides valuable insights for informing future research and development in the field. Kolekar et al. [1] introduced explainable AI (XAI) in scene understanding for autonomous vehicles, focusing on navigating the chaotic traffic landscape of Indian roadways. Through a systematic literature review, they address the challenge of scene understanding in dynamic traffic environments, evaluating AI solutions for transparency and explainability. Critically examining limitations of conventional AI models, Kolekar et al. propose integrating the U-Net model with inception augmented with the Gradient-weighted Class Activation Mapping visualization technique as a solution. Their review offers a comprehensive understanding of current state-of-the-art techniques, enhancing reliability and interpretability of AI-driven scene understanding systems for self-driving cars in challenging scenarios. Kuutti et al. [7] surveyed deep learning applications for controlling self-regulating vehicles, exploring various algorithm approaches in perception, decision-making, planning, and control tasks. Investigating challenges and efficacy of different approaches, they also assess implications of deep learning on performance, scalability, robustness, and security of Autonomous Vehicle Control Systems.

Marius et al. [8] stressed the importance of obtaining and interpreting the annotated datasets for better comprehension of the semantic urban scenes. They introduced the Cityscapes dataset to foster the development of algorithms for performing the task. Cityscapes is a dataset for detecting objects in metropolitan scenes. This dataset is a large body of high-definition pictures that have been labelled using a semantic definition as well as drivable soils. DFE-AVD was developed using a multistep learning approach to enhance the identification of cars. The approach cooperated with multiple deep CNN models to obtain improved performance in an inventive ensemble system. Research on systemization with the UNet segmentation architecture and the MobileNet v2 encoder has shown promising results [9]. Fan et al. [10] devised a real-time scene parsing network using a modified MobileNetv2 encoder and efficient space module propagation, achieving 71.4 % mIoU on cityscapes at 67 FPS inference speed. TernausNet, by Iglovikov and Shvets [11], utilizes pre-trained VGG19 encoders and decoders with U-Nets to achieve 84.5 % accuracy on the ISBI Challenge for segmentation. The authors probed human-centric mechanisms, causality, spatial and temporal dependencies and the usual rules within the integrated actions frameworks. Still, they met restrictions in common sense reasoning, model decision making and safety necessities [12]. Yuan et al.'s OCNet [13] introduces a novel contextual data aggregation module that enhances the segmentation of complex scenarios by aggregating multilevel features in a U-shaped route. They propose a Layerwise Integration approach using a dual path coordination mechanism between compact decoders and ResNet Encoders for accurate and efficient scene parsing. Gupta et al. [14] conducted a survey tracing the evolution of traditional computer vision approaches and the success of deep neural networks in object detection, emphasizing the importance of datasets, benchmarking measures, and simulation settings in research development across various models, designs, challenges, and new domains.

Dhillon and Verma's [15] review paper is an example of a deeper investigation of convolute neural networks in object identification. The review outlines critical advances and their potential in object recognition and multi-object pursuit but also defines difficulties and research possibilities in the future. The authors illustrate that the possible utilization of Grad-CAM can aid in mitigating the divide between complex models and simple interpretations by humans, as it is crucial for developing transparent and interpretable AI systems in the legal sector. Need for wide-scale adoption [16]. In 2020, Desai and Ramaswamy [17] suggested an ablative technique highlighting critical areas on input images while eliminating the requirement for gradient-based localization procedures. They expanded interpretability and trust in AI methods while acknowledging current approaches and shortcomings through a gradient-free method. Delibas and Cetin [18] analyzed the use of deep learning methods to detect buildings, integrating the initial blocks to improve the UNet architecture, increasing multiscale feature representation, and enhancing the performance of urban planning and disaster management.

When using vehicle datasets to assess recognition and segmentation activities, Singh et al. [19] emphasized the significance of precise detection for the effectiveness and safety of autonomous driving. They emphasize the need for a coherent evaluation framework. Ding et al. [20] examined the propagation of boundary features for scene- aware segmentation, highlighting the importance of accurate border detection to deliver correct results. An improved approach was proposed that would improve the aggregation of features and the accuracy of delineations. He et al. [21] stressed the importance of contextual information in semantic segmentation algorithm performance. They introduced an Adaptive Pyramid Context Network, where the pyramid context is adaptable at every level, to enhance segmentation performance in various scenarios. Xu et al. [22] conducted a literature review on road segmentation using loss functions with remotely detected information. They examined the effectiveness of loss functions in addressing imbalanced class distribution and noise annotation, providing insights into network optimization to increase accuracy. Khayyam et al. [2] reviewed AI literature on IoT applications for autonomous cars, advocating for these technologies' essential role in real-time data transfer and decision-making. They explored methods for enhancing navigation, safety, and traffic flow. In another study, various deep learning techniques, including the VGG16 and VGG19 architectures, are utilized for different applications such as face liveness detection, as experimented on in the paper [[23], [24]].

Inception modules enhance brain tumor segmentation by enhancing efficiency and accuracy. The authors of a paper [25] examine existing methods, identifying limitations such as sensitivity to noise and computational complexity. They proposed an approach involving preprocessed MRI images and feature extraction to address these challenges. Additionally, [26] reviewed deep learning techniques for medical imaging, focusing on convolutional neural networks, transfer learning, and attention mechanisms in semantic segmentation. While existing implementations have demonstrated notable achievements, there remains room for enhancing performance. Although current CANet model implementations have shown noteworthy mIoU performance, reaching 0.5485 for validation data, there is an opportunity for further improvement by integrating the CANet model. Hence, in this research, we propose a novel architecture that integrates the LinkNet and Unet models with the foundational Chained Context Aggregation Network (CANet) model to address this. By combining these distinct models, we anticipate performance improvements. The improvement can be measured with the mean Intersection over Union (IoU), a crucial parameter in the assessment of semantic segmentation.

## Method details

One of the most important tasks in computer vision is semantic segmentation, aimed at identifying and classifying every pixel in the image. Many applications, ranging from self-driving cars to medical image analysis, are performed by this mechanism. Traditional methods usually require large annotated datasets to learn from, and creating them is expensive and time-consuming. The aspect of autonomous vehicle scene interpretation aspect is semantic segmentation, which is achieved using the CANet architecture deep learning model. This architecture approach consists of a Multiscale Contextual Module with parallel branches, which acquires contextual knowledge at three scales: interdisciplinary, global, and local [5]. When merged with items and road markings with/linked with their background, this information is used by CANet to predict the relations among these components in the complex driving context. The scene components are classified by the CANet using hypothetical knowledge to allow the autonomous cars to see and travel the environment comfortably and safely. The CANet architecture has demonstrated improved performance in segmenting city driving situations to increase scene comprehension capabilities for autonomous vehicles.

## Data description

**Fig. 1:**
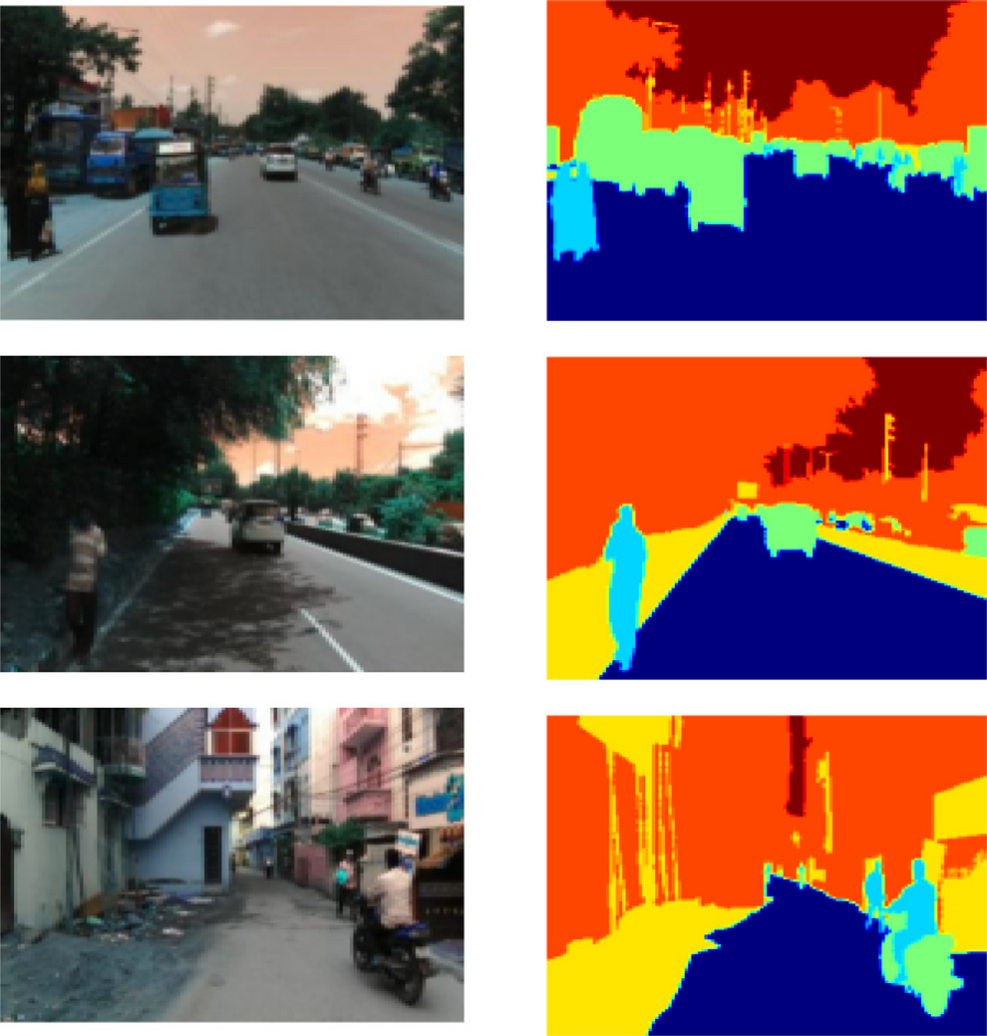
Original images with ground truth from IDD-Lite dataset.

The proposed architecture's performance was assessed using the IDD-Lite dataset [1]. The IDD dataset is an innovative dataset designed to help with road scene understanding in unstructured environments, which are typically characterized by clearly defined infrastructure (lanes, for example), a few precise categories for traffic participants, slight variation in the appearance of objects or backgrounds, and scrupulous observance of traffic laws. A lite version of the dataset, IDD-Lite, offers the same number of statistics as IDD and is accessible in cases where resources are restricted [1]. IDD-Lite dataset consists of seven classes: non-drivable, living things, drivable; wheeler, auto-rickshaw, large vehicle; barrier, structures, construction, vegetation and sky. This dataset comprises 1380 train images and 204 test images at 320 × 227 pixel resolution, which is about 50 MB. It comprises two files: leftlmg8bit and gtFine. gtFine has the val and train folders, whereas leftlmg8bit contains the val, train, and test folders. Fig. 1 illustrates some of the sample images of the IDD-Lite dataset.

### Computational requirements

The proposed model was trained on a system with the following specifications: 64-bit Windows operating system, AMD Ryzen 5 2.10 GHz processor, 8 gigabytes of RAM, and access to Google Colab Pro, which provides 51 gigabytes of RAM and 15 gigabytes of GPU resources. The Google Colab pro-environment was used to train the model, which required a T4 GPU and 51 GB RAM.

### Architecture of proposed method

**Fig. 2:**
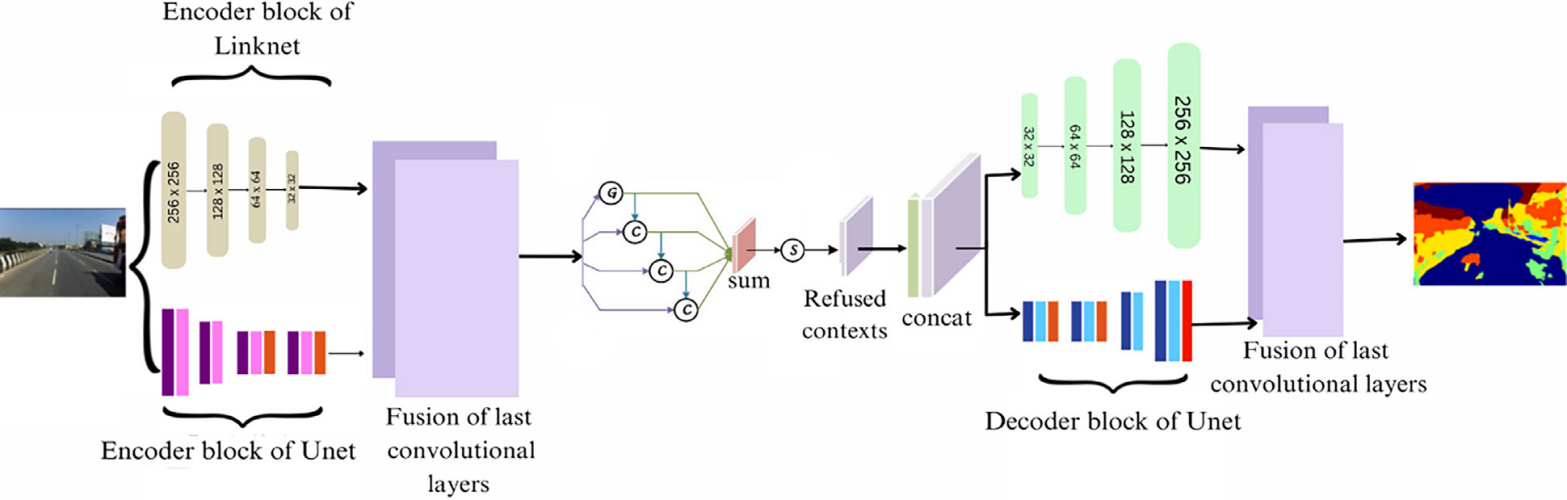
Architecture of the proposed method.

This work proposed an integrated architecture presented in Fig. 2 that combines UNet, Linknet and the CANet. The encoder components of the U-Net and Link-Net models, which comprise the convolutional layers and blocks, were used in lieu of the convolutional layers of the original CANet model. The encoder method reduces the feature map resolution to obtain the most compelling features from the source image [3]. Feature maps of the last convolutional layers of the UNet and LinkNet models are merged and fed the result into the next architecture layer. Further, the decoder parts of both models are included for concatenation. The last convolutional layers of the decoder part of both models are fused and obtained the final output.

**Fig. 3:**
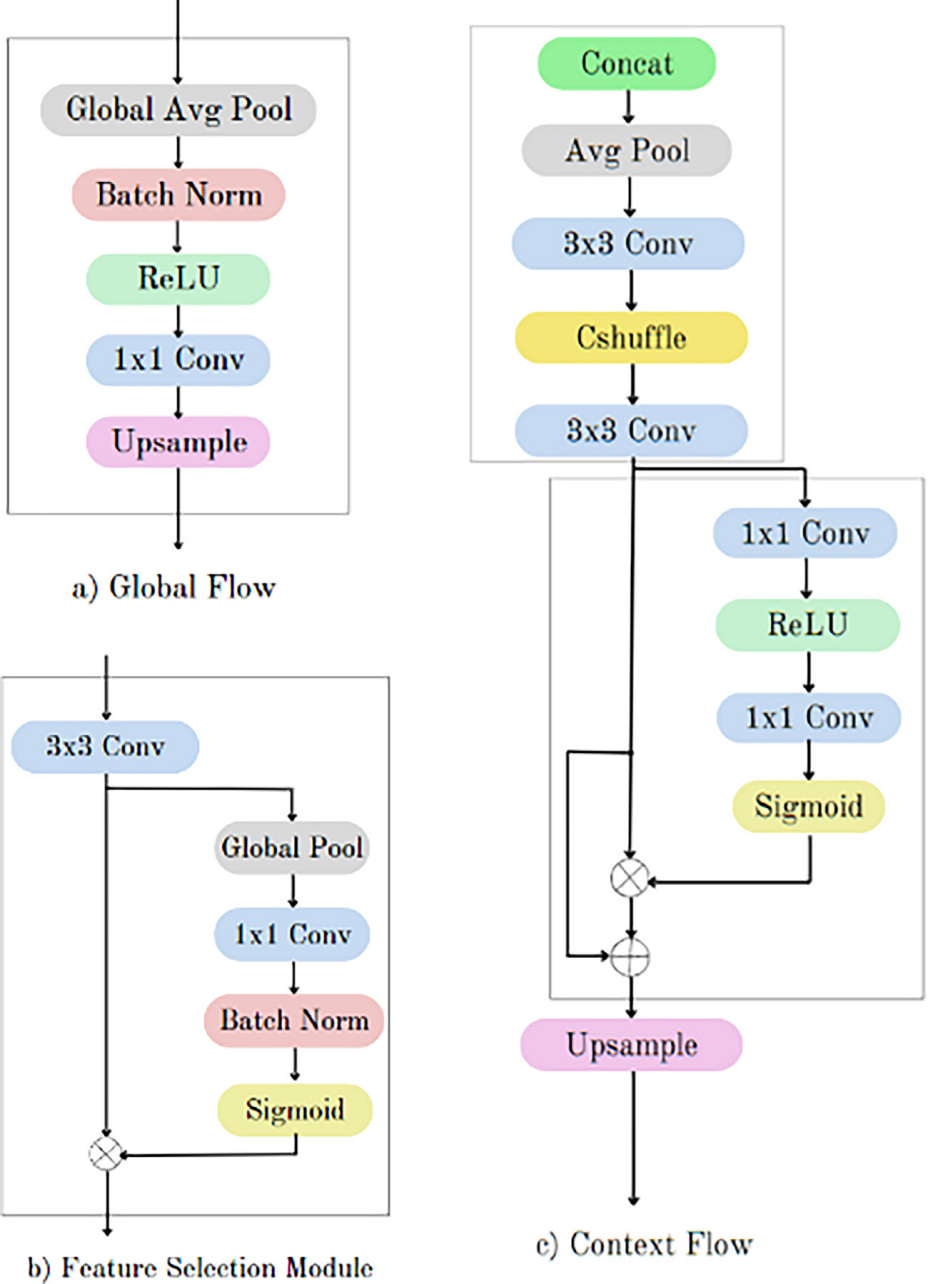
Flow diagram of global flow, feature selection module and context flow.

The output of the fusion of the encoder parts of UNet and LinkNet models is fed as an input into the global flow, which outputs a global feature map with all of the image's metadata. Global pooling features may serve as a dependable indication to differentiate confused objects, as demonstrated by practices [[27], [28], [29], [30]]. First, the Batch normalization layer, the ReLU activation function, and the 1 × 1 convolutional layer are applied. Then, global average pooling is applied to the shared feature maps of the backbone network. Fig. 3(a) depicts the flow diagram of the global flow component of the overall architecture.

The final convolution block's output and the global flow's output are extracted and concatenated as a single unit in the context flow module, which is part of the context fusion module. Following that, convolutional layers and average pooling are used. Finally, the following process (1) is carried out in the context refinement module before up-sampling. Fig. 3(c) depicts the flow diagram of the context flow component of the overall architecture.

(1) Y=(A⊗B)⊕A

where, *A* = output from the 3 × 3 conv layer of context flow

*B* = output from the sigmoid layer of context flow

⊗ represents the tensor (or Hadamard) product

⊕ represents the element-wise addition.

The Feature Selection Module (FSM) receives input from the output feature maps of a convolutional block and the context-aware feature map from the context flow layer. After concatenating these feature maps along the channel axis, gating masks for each feature map are calculated using two convolutional layers with sigmoid activation functions. The final output is then obtained by applying these masks to the input feature maps and adding the resultant features to the original feature maps. Fig. 3(b) depicts the flow diagram of the feature selection module of the overall architecture.

**Fig. 4:**
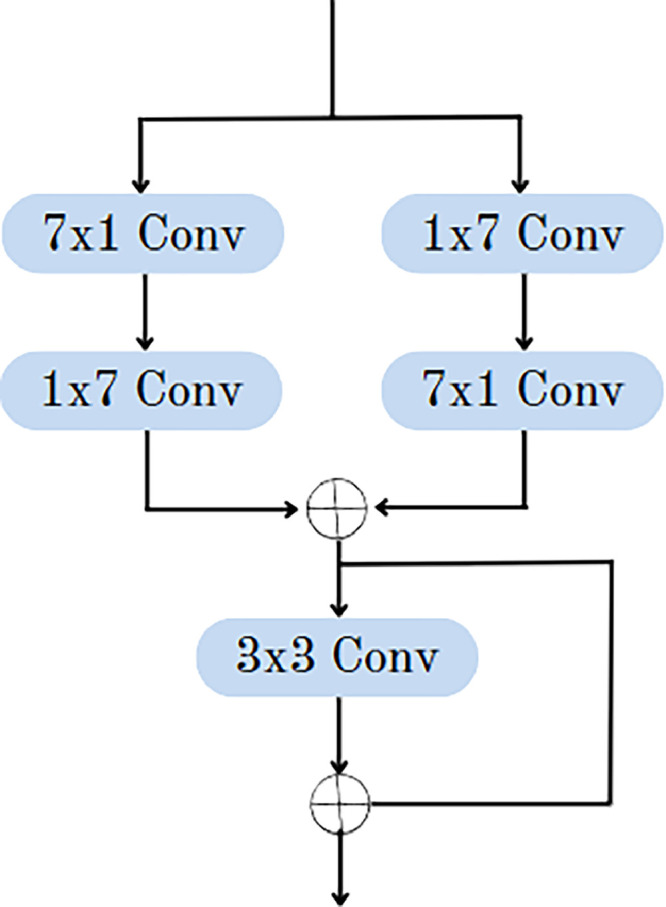
Flow diagram of AGCN.

The first convolutional layer of the encoders from both the U-Net and Link-Net models will be combined to form one of the convolution blocks, which will be the input for the AGCN module. All of the convolutions in AGCN will have the same padding and stride, equal to one. The input shape and the output shape will be identical in this case. Fig. 4 represents the flow diagram of the AGCN component of the complete architecture. The collective findings of these studies illustrate the progression of semantic segmentation, as it tackles challenges and enhances its practicality.

## Method validation

The various essential evaluation metrics demonstrated that the model trained on the semantic segmentation data performed very well. The semantic segmentation models for individual CaNet, LinkNet and UNet models are evaluated using six metrics: intersection over union, loss, F-score, specificity, sensitivity, and accuracy [31].

Mean IoU, also known as the Jaccard index [32], calculates the average intersection over union across all segmentation masks, where IoU measures the overlap between the predicted and ground truth masks.

(2) $$\text{Specificity}=\frac{True\;Negative}{True\;Negative+False\;Positive}$$

(3) $$\text{Sensitivity}=\frac{True\;Positive}{True\;Positive+False\;Negative}$$

(4) $$F1\;\text{Score}=2^{*}\left(\frac{Precision*Recall\;}{Precision+Recall\;}\right)$$

(5) $$\text{Precision}=\frac{True\;Positive}{True\;Positive+False\;Positive}$$

These indicators assess how well models in the IDD Lite dataset can distinguish between classes, for example, pedestrians, cars and roads. Better segmentation performance is indicated by higher specificity, sensitivity, F1 and mean IoU scores. The models demonstrated excellent segmentation accuracy, excellent IoU metrics, and low training and validation losses for IDD Lite data. However, there is still room for improvement using different approaches.

The findings were validated by experimenting with the proposed CANet architecture, which combined the Linknet and Unet encoders and decoders. It aids the model in learning more precise representations of features and patterns by using the whole training dataset. The proposed model's outcomes are contrasted with the most recent models, such as SegNet, UNet-ResNet34, E-Net, UNet-ResNet18, Inception U-Net, and U-Net [1]. Table 1 depicts the performance evaluation of the proposed CANet architecture for the training and the validation dataset. Table 1 shows that the mean IoU for the proposed CANet architecture for the training and validation dataset for 50 epochs is 0.7864 and 0.7053, respectively. The accuracy, specificity, F1 score, dice coefficient, and sensitivity of the proposed CANet model for the IDD-Lite validation dataset are 0.8477, 0.9795, 0.8306, 0.9757 and 0.8227, respectively.

**Table 1:** Performance evaluation for the proposed CANet architecture.

Model	Dataset	Mean IoU	Accuracy	Specificity	Sensitivity	F1 score	Dice Coefficient
Proposed CANet	Training	0.7864	0.9152	0.9895	0.8998	0.9891	0.8792

Table 2 compares the various state-of-the-art models with the proposed CANet model with Unet and Linknet on the validation data of the IDD-Lite dataset. Semantic segmentation models' efficacy is assessed using the mean IoU measure [1]. The suggested CANet model has a mean IoU score of 0.7053, higher than all other advanced segmentation models in Table 2. Table 3 presents the mean IoU values of the state-of-the-art segmentation models and the proposed CANet model.

**Table 2:** Performance evaluation for the proposed CANet architecture.

Model	Mean IoU	Accuracy	Sensitivity	Specificity	F1 Score
E-Net	0.566	0.9321	0.8669	0.9395	0.7229
UNet-ResNet34	0.6174	0.9398	0.8617	0.9484	0.7635
SegNet	0.3076	0.8971	0.8896	0.8975	0.4705
UNet-ResNet18	0.5981	0.9356	0.8472	0.9469	0.7485
U-Net	0.6031	0.9203	0.8534	0.9500	0.7056
Inception U-Net	0.622	0.958	0.728	0.975	0.740
Proposed CANet model	0.7053	0.8477	0.8306	0.9795	0.9757

**Table 3:** Comparison of the proposed model with the SOTA models based on MIo.

Model	Proposed model	U-Net	Inception U-Net	UNet-ResNet18	SegNet	E-Net	UNet-ResNet34
MIoU	0.7053	0.6031	0.622	0.5981	0.3076	0.566	0.6174

**Fig. 5:**
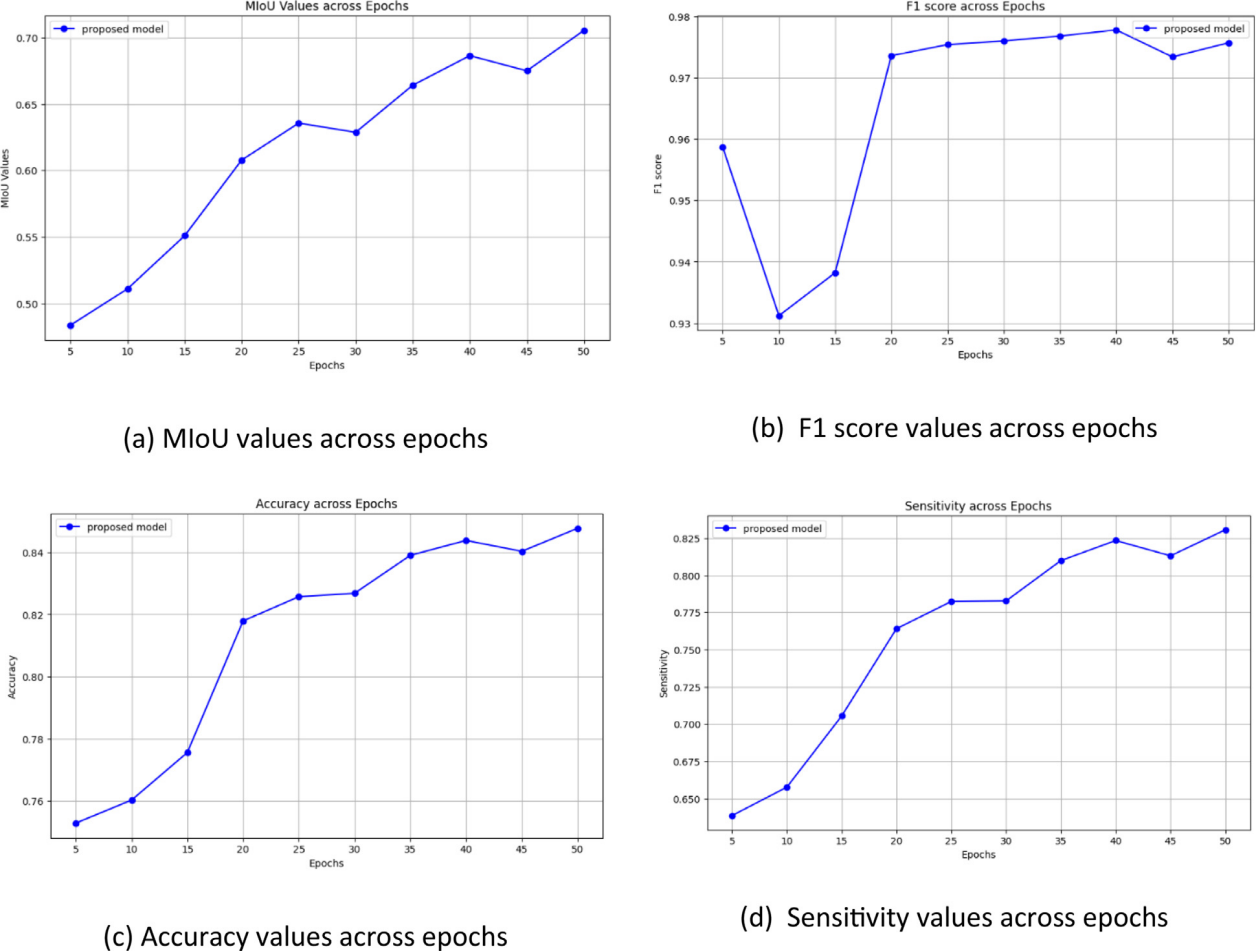
Values of Performance metrics across epochs.

**Fig. 6:**
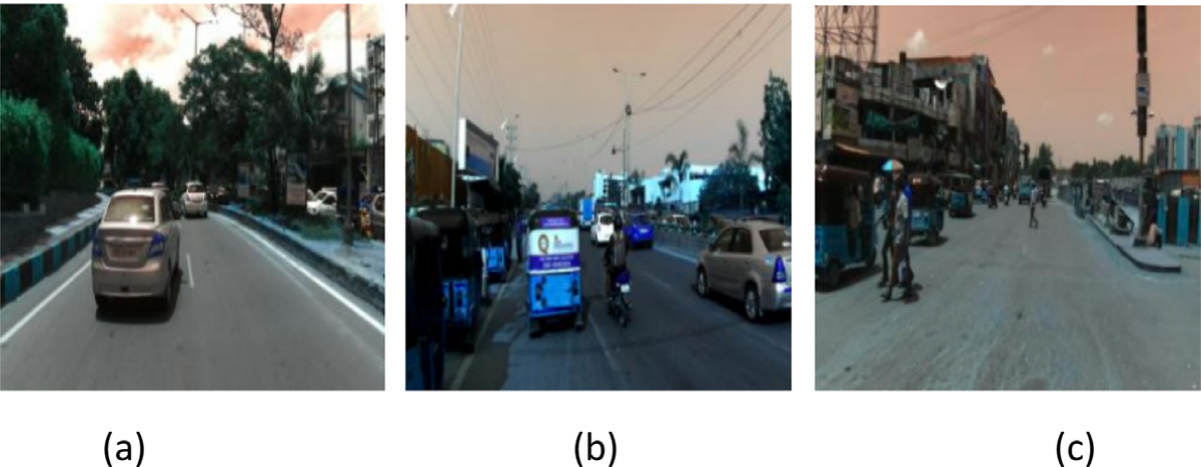
Sample input images from the original IDD-Lite Dataset.

**Fig. 7:**
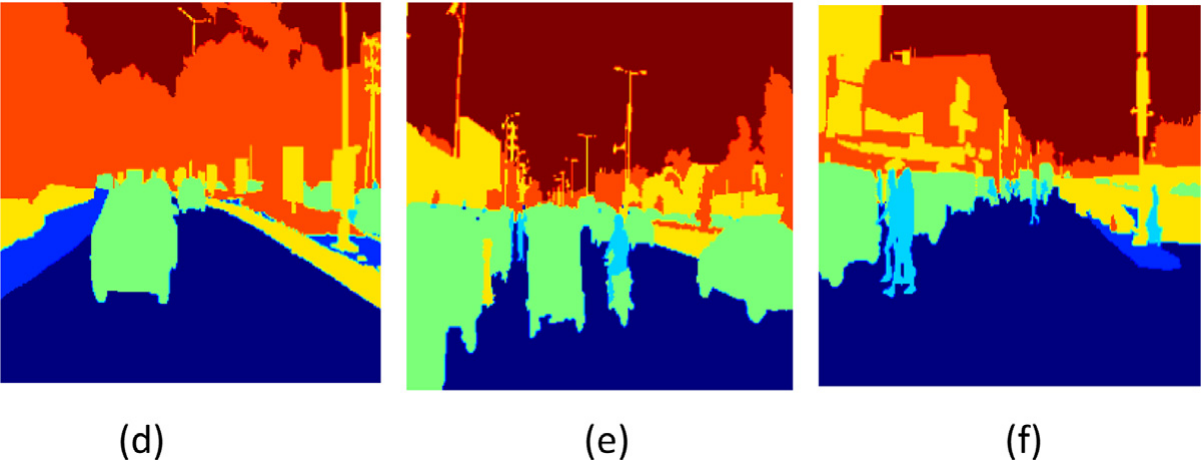
Ground truth images of respective sample input.

**Fig. 8:**
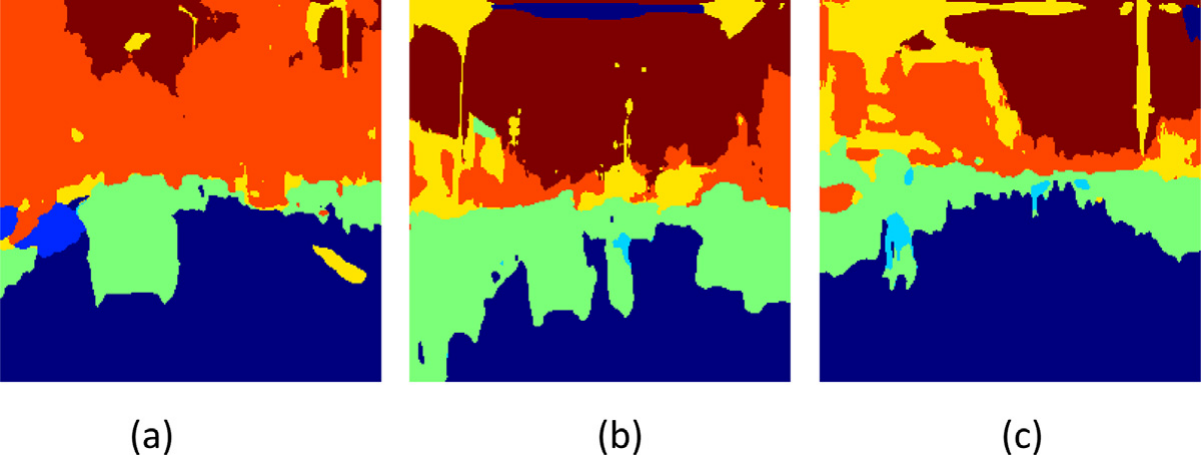
Segmented image outputs of respective input images for the proposed model.

Fig. 5(b) displays the accuracy results for the suggested model over 50 epochs. The whole pixel-level accuracy of semantic segmentation predictions is measured by accuracy. You can assess convergence and stability using the graphic as the optimization progresses. The Mean Intersection over Union (MIoU) for the suggested architecture throughout 50 epochs is shown in Fig. 5(a). Besides accuracy, MIoU is a crucial performance statistic for semantic segmentation since it measures the difference between predictions and reality for each class individually. Information on the consistency of appropriate semantic class discovery can be obtained by tracking the model's sensitivity over time. The models' per-epoch sensitivity values during a 50-epoch period are displayed in Fig. 5(d). More significant numbers indicate a higher ability to identify a class precisely. Lastly, the F1 scores for 50 epochs are displayed in Fig. 5(c). The F1 measure considers recall in addition to accuracy. Analyzing balanced progress in precisely predicting pixels of a given class while gathering all of that class's pixels is examined by F1 overtraining. Combined, these four graphs comprehensively evaluate numerous segmentation performance elements during each model's iterative optimization process. Fig. 6, Fig. 7, Fig. 8 show the input images, ground truth images and their respective segmented output image of the proposed CANet architecture.

## Discussion

The Intelligent Transport System (ITS) relies heavily on road scene segmentation for safer vehicle navigation and accurate environmental interpretation. It is easier for autonomous vehicles to operate safely in heavily congested places when they understand their surroundings better and can interpret the behaviour of nearby vehicles. To facilitate the study, companies such as LYFT, WYMO, and Argoverse make their datasets openly accessible. These datasets were gathered in developed nations with organized transportation networks. IDD published the dataset for the unstructured environments where the traffic is unpredictable, such as in developing countries like India.

The research implemented modified CANet architecture, including the U-Net and Link-Net models. The encoders and decoders of both the U-Net and Link-Net models are used in the basic CANet architecture, replacing the convolutional layers of CANet, giving better results. The other existing models were then compared to the suggested model. The suggested model outperformed the previously described models in terms of results. Table I presents the proposed CANet architecture's performance metrics.

The suggested model's accuracy and mean IoU value for the training dataset are 0.9152 and 0.7864, respectively. Based on the mean IoU values for the validation dataset, the suggested model is compared with other existing models and outperformed all other models. The state-of-the-art models used for the comparison include Inception U-Net, UNet-ResNet34, Segnet, UNet-ResNet18, E-Net, and U-Net. The comparative analysis of all the models with the proposed model is represented in Table II. The more values for the current models are 0.622, 0.6174, 0.3076, 0.5981, 0.566, and 0.6031, respectively. At the same time, the mean IoU value for the proposed model is 0.7053. The proposed model outperforms the state-of-the-art semantic segmentation models based on the mean IoU score. Table III compares the mIoU values of all the SOTA models with the proposed model. The mean IoU was also calculated for the test data with a value of 0.6831. Better performance is achieved in the suggested model by substituting the encoder and decoder of the U-Net and Link-Net for the convolutional layers of the CANet model.

## Limitations

One limitation of the proposed CANet model is its extensive architecture with many layers, increasing computational time. This is attributed to using encoder and decoder components from the U-Net and Link-Net architectures within the CANet framework. Consequently, the overall convolutional layers are augmented, exacerbating the computational burden.

## Ethics statements

Not Applicable

## CRediT author statement

**Data curation:** B.P. and S.D.; **Methodology:** S.D., B.P., R.G. and S.S.K; **Writing-original draft:** S.D., B.P., R.G., and A.C.; **Supervision:** S.C.K., S.G. and S.S.K.; **Visualization:** B.P., A.A. and S.D.; **Validation:** B.P., A.A., S.C.K.; **Project administration:** S.C.K. and B.P and A.A.; **Writing-review and editing:** S.C.K., B.P., S.D., R.G., and A.A.; **Resources:** S.C.K. and S.K; **Conceptualization:** S.C.K. and S.S.K.

## Funding

This research was funded by the Centre for Advanced Modelling and Geospatial Information Systems (CAMGIS), Faculty of Engineering and IT, University of Technology Sydney and Researchers Supporting Project, King Saud University, Riyadh, Saudi Arabia, under Project RSP2024 R14.

## Declaration of competing interest

The authors declare that they have no known competing financial interests or personal relationships that could have appeared to influence the work reported in this paper.

## Data Availability

Data will be made available on request.
